# Vitamin D in the Prevention of Aromatase Inhibitor–Induced Musculoskeletal Symptoms: Is It Ready for Practice?

**Published:** 2012-07-01

**Authors:** Jeannine Brant

**Affiliations:** From Billings Clinic, Billings, Montana

Breast cancer is the most common malignancy in women. In 2012, approximately 226,870 new cases of breast cancer will be diagnosed in the United States (American Cancer Society, 2012). Depending on tumor characteristics and stage of disease, breast cancer treatment includes surgery, radiation therapy, chemotherapy, biologic therapy, and hormonal therapy.

Aromatase inhibitors (AIs), one type of hormonal therapy, are indicated in the treatment of estrogen receptor–positive breast cancer in postmenopausal women (National Comprehensive Cancer Network, 2012). Although tamoxifen was traditionally used in the treatment of hormone receptor–positive breast cancer, the randomized controlled ATAC (Arimidex, Tamoxifen, Alone or in Combination) trial (N = 9,366) comparing tamoxifen with anastrozole (an AI) plus tamoxifen indicated that anastrozole reduced the risk of tumor recurrence in postmenopausal women with localized breast cancer by 40%. Unfortunately, the use of anastrozole and other AIs is associated with musculoskeletal symptoms, including an increased risk of bone fractures and musculoskeletal pain (Howell et al., 2005). Symptoms can affect both adherence and quality of life (Chlebowski, 2009).

A recent study by Rastelli and colleagues (2011) suggests that vitamin D supplementation can decrease aromatase inhibitor–induced musculoskeletal symptoms (AIMSS). This article will discuss the problem of musculoskeletal symptoms with AI use, treatment strategies currently suggested in the literature, recent results of the vitamin D study by Rastelli and colleagues, and implications for advanced practitioners.

## Aromatase Inhibitor–Induced Musculoskeletal Symptoms

Aromatase inhibitors can cause a multitude of musculoskeletal symptoms. While many aging women without cancer experience arthralgias and myalgias in their daily lives, clinical trials report that approximately 33% of women taking AIs experience an increase in arthralgias (Howell et al., 2005). It is likely that the overall prevalence of arthralgias is underestimated; as ATAC’s primary objective was breast cancer response, adverse effects may have been underreported (Sestak et al., 2008). Another trial examined musculoskeletal symptoms in postmenopausal women with nonmetastatic breast cancer who were about to start aromatase inhibitor therapy (Napoli et al., 2010). Arthralgias and myalgias were found in 61.3% and 43% of patients, respectively.

Little information is known about risk factors for musculoskeletal symptoms and the course of disease. Studies suggest that obese women and those who received prior chemotherapy may be at greater risk for arthralgias (Sestak et al., 2008). Vitamin D deficiency was noted in the majority of women with AIMSS (Napoli et al., 2010). Studies have suggested that for some women, arthralgias improve within 6 months of initiation, even while undergoing AI therapy. When arthralgias significantly impact quality of life, rotating to an alternative AI often results in a decrease in joint pain and swelling (Briot, Tubiana-Hulin, Bastit, Kloos, & Roux, 2010; Fontaine et al., 2008). In addition to arthralgias, women undergoing AI therapy can experience carpal tunnel syndrome. Estrogen also contributes to bone mineral density (BMD). A major adverse effect related to AIs is a decrease in bone density, resulting in osteopenia and osteoporosis (Howell et al., 2005).

## Management of AIMSS

Few trials have investigated interventions to manage arthralgias and other musculoskeletal symptoms in women taking AIs. One expert panel recommended selective COX-2 and nonselective nonsteroidal anti-inflammatory drugs, based on treatment of joint pain unrelated to AI administration (Coleman, Body, Gralow, & Lipton, 2008). One study compared sham acupuncture (superficial needle insertion at nonacupoint locations) with true acupuncture (full body/auricular acupuncture and joint-specific point prescriptions) in 38 women with AIMSS. True acupuncture significantly improved joint pain and stiffness (*p* < .001; Crew et al., 2010).

As vitamin D plays an important role in bone health, supplementation is recommended for all postmenopausal women. Vitamin D has also sparked some interest for the management of AIMSS as deficiency was noted in many arthralgia sufferers who were taking AIs. Trials using standard (400 IU per day) and high doses (50,000 IU per week) of vitamin D_3_ in postmenopausal women not taking AIs showed no improvement in arthralgias (Chlebowski, 2009). A small (N = 50) prospective single-arm study examined the effect of high-dose vitamin D on musculoskeletal symptoms in breast cancer patients receiving the AI letrozole. Relief of joint pain was reported by 23% of patients, although this was not statistically significant (Khan, O’Dea, & Sharma, 2010).

A larger trial aimed to establish a level of vitamin D to prevent or minimize arthralgia in a cohort of 290 women starting AI therapy. While all women received standard doses of vitamin D_3_ (800 IU) with calcium, those with a baseline vitamin D level less than 30 ng/mL also received 16,000 IU of vitamin D_3_ every 2 weeks. Even with supplementation with higher doses, 50% of the women failed to reach adequate vitamin D levels at 3 months. Joint pain was attenuated in those who reached vitamin D levels of at least 40 ng/mL (Prieto-Alhambra et al., 2011).

## The Rastelli Vitamin D Trial

The most notable study to date is a double-blind placebo-controlled randomized phase II trial using vitamin D for AIMSS. Patients (N = 60) were stratified according to baseline vitamin D (25-hydroxyvitamin D [25-OHD]) level (Rastelli et al., 2011). Those with moderately low levels, between 20 and 29 ng/mL, were randomized to receive either high-dose vitamin D supplementation (50,000 IU) weekly for 8 weeks then monthly for 4 months, or placebo. Those with low levels, between 10 and 19 ng/mL, were randomized to receive high-dose vitamin D for 16 weeks and then monthly for 2 months, or placebo. Symptoms were assessed at baseline and at 2, 4, and 6 months using the Brief Pain Inventory–Short Form (BPI), the Fibromyalgia Impact Questionnaire (FIQ), and the Health Assessment Questionnaire–Disability Index. Investigators also assessed BMD at baseline and at 6 months.

Results indicated that pain was significantly decreased in the high-dose vitamin D groups compared to the placebo groups according to the FIQ (*p* = .0045) and several measures on the BPI: worst pain (*p* = .04), average pain (*p* = .0067), pain severity (*p* = .04), and interference (*p* = .034). When each of the groups were analyzed separately, those patients who received the vitamin D for 16 weeks had more statistically significant improvement in symptoms. In terms of BMD, the femoral neck decreased in the placebo group, but no change was noted in the high-dose vitamin D group (*p* = .06).

No significant gastrointestinal adverse events were reported in the high-dose vitamin D groups. However, this was not a primary variable in the study, and specific measures of adverse events were not discussed. Five patients dropped out of the study due to hypercalcemia, four of whom were in the high-dose vitamin D groups (Rastelli et al., 2011). Gastrointestinal symptoms such as nausea and diarrhea have been reported with low-dose vitamin D supplementation (doses less than 1,000 IU/day). Hypercalcemia and increased renal disease can also ensue (Chlebowski, 2009). Further studies are needed to determine adverse events related to high-dose vitamin D in women with breast cancer who are receiving AIs.

## Future Directions

While research suggests that the use of high-dose vitamin D in the management of AIMSS is effective, additional studies are needed to determine exact dosing strategies, monitoring guidelines, and adverse effects. Two additional clinical trials are underway to investigate this problem: (1) A Randomized Trial to Evaluate the Benefit of High Dose Vitamin D_3_ on Aromatase Inhibitor Letrozole-Associated Musculoskeletal Symptoms and Fatigue (The VITAL Trial) and (2) Vitamin D_3_ Effects on Musculoskeletal Symptoms With Use of Aromatase Inhibitors (National Institutes of Health, 2012). Although the first trial is closed but ongoing, the second trial is currently accruing patients.

## Implications for Advanced Practitioners

Advanced practitioners are on the front lines of symptom management. For those in breast cancer care, arthralgias are a common complaint among women and have a significant impact on quality of life. Some women are impacted to the degree that they are no longer adherent to therapy. The results of this study may prompt some practitioners to move this research directly into practice by prescribing and recommending high doses of vitamin D for patients with AIMSS; however, additional studies are needed to determine the overall safety and efficacy in the long-term use of high-dose vitamin D in women on AIs. Questions remain such as how often to dose, how long to continue treatment, what types of measures should be used to monitor patients, how often to monitor patients, and when to discontinue treatment due to adverse effects such as hypercalcemia.

Vitamin D is increasingly recognized for its positive health benefits aside from managing arthralgias in patients on AIs. Prevention of autoimmune disease, cancer, cardiovascular disease, depression, dementia, infectious diseases, and musculoskeletal decline are all mentioned in the literature (Haines & Park, 2012). Some patients are at high risk for low 25-OHD levels, especially those individuals from northern communities who have limited sun exposure. Lack of vitamin D in the diet, liver and kidney disease, malabsorption, and medications such as phenytoin can also contribute to low levels. Advanced practitioners can take an active role in monitoring 25-OHD levels in high-risk patients and replacing vitamin D as needed to maintain normal levels (30–74 ng/mL). Monitoring for side effects is also important to ensure patient safety and well-being. Clinicians should be aware of the adverse effects of vitamin D and recognize these effects when recommending or prescribing vitamin D. While it is rare, hypervitaminosis can occur (Haines & Park, 2012; see Table 1).

**Table 1:**
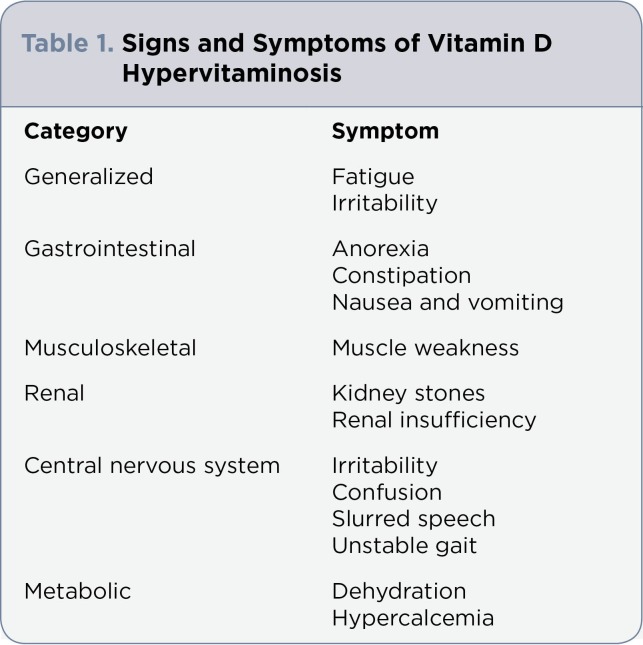
Signs and Symptoms of Vitamin D Hypervitaminosis

It is unknown whether replacement of vitamin D to normal levels will alleviate AIMSS, but other benefits alone may improve quality of life for patients and prevent other health complications. Advanced practitioners should continue to keep watch for additional studies in the literature that will more thoroughly answer this question about vitamin D use for AIMSS.
